# A computational fluid dynamics approach to determine white matter permeability

**DOI:** 10.1007/s10237-019-01131-7

**Published:** 2019-02-20

**Authors:** Marco Vidotto, Daniela Botnariuc, Elena De Momi, Daniele Dini

**Affiliations:** 1 Department of Electronics, Information and Bioengineering, Politecnico di Milano, 20133 Milan, Italy; 2 Department of Mechanical Engineering, Imperial College London, London, SW7 2AZ UK; 3 Faculty of Science, University of Lisbon, Campo Grande, 1149-016 Lisbon, Portugal

**Keywords:** Convection-enhanced delivery, Hydraulic permeability, Representative volume element, White matter

## Abstract

Glioblastomas represent a challenging problem with an extremely poor survival rate. Since these tumour cells have a highly invasive character, an effective surgical resection as well as chemotherapy and radiotherapy is very difficult. Convection-enhanced delivery (CED), a technique that consists in the injection of a therapeutic agent directly into the parenchyma, has shown encouraging results. Its efficacy depends on the ability to predict, in the pre-operative phase, the distribution of the drug inside the tumour. This paper proposes a method to compute a fundamental parameter for CED modelling outcomes, the hydraulic permeability, in three brain structures. Therefore, a bidimensional brain-like structure was built out of the main geometrical features of the white matter: axon diameter distribution extrapolated from electron microscopy images, extracellular space (ECS) volume fraction and ECS width. The axons were randomly allocated inside a defined border, and the ECS volume fraction as well as the ECS width maintained in a physiological range. To achieve this result, an outward packing method coupled with a disc shrinking technique was implemented. The fluid flow through the axons was computed by solving Navier–Stokes equations within the computational fluid dynamics solver ANSYS. From the fluid and pressure fields, an homogenisation technique allowed establishing the optimal representative volume element (RVE) size. The hydraulic permeability computed on the RVE was found in good agreement with experimental data from the literature.

## Introduction

The most common brain malignant tumours, glioblastomas multiforme (GBMs), leave patients a median overall survival rate ranging from 12 to 18 months, as reported in Mehta et al. (2015). Moreover, despite affecting only 6 in 100,000 people, the treatment cost in Europe in 2010 was about 5.2 billion Euro (Olesen et al. 2012). Conventional treatment options such as surgery, chemotherapy and radiation have not proved themselves as decisive, despite being highly aggressive for the patients (Crawford et al. 2016). Therefore, Bobo et al. (1994) introduced a new technique, namely CED, which has shown encouraging results with recurrent glioblastoma in the last 20 years (Crawford et al. 2016). Indeed, it allows overcoming the main obstacle to pharmaceutical treatment of tumour, the blood–brain barrier, by injecting a therapeutic agent under positive pressure directly into the parenchyma.

A key aspect to reach good results is the ability to predict, in the pre-operative phase, the distribution of the drug inside the tumour (Raghavan et al. 2006, 2016). This would allow planning the infusion point and the flow rate to optimise the treatment. Several studies have been conducted in the last 15 years proposing numerical models which were based on different assumptions (Ehlers and Wagner 2013; Støverud et al. 2012; Linninger et al. 2008; Kim et al. 2012; Sarntinoranont et al. 2006; Chen and Sarntinoranont 2007; Morrison et al. 1999; Raghavan et al. 2006; Raghavan and Brady 2011; Smith and García 2009). Nonetheless, the cerebral tissue complex structure has represented a formidable challenge to modelling, and more studies should be conducted to reach a satisfying level of accuracy. As suggested by Ehlers and Wagner (2013) and Støverud et al. (2012), this could be due to the fact that the constitutive parameters which are used in the models vary significantly across the scientific literature (up to three orders of magnitude). Therefore, in this paper, we aimed to shed light on the hydraulic permeability which is one of the key parameters affecting CED outcomes. Indeed, it drives the convective flux through the brain thus determining the pharmaceutical agent ability to spread within the cancerous tissue.

The brain could be divided in three main components characterised by different properties: cerebrospinal fluid (CSF), grey matter and white matter. The CSF can be found in all the empty spaces within the skull thus comprising the gap between the brain and the skull, the ventricles and the ECS. The grey matter consists of neuron cell bodies which are highly packed making the tissue very dense. In contrast, the white matter can be found in the inner part of the brain and presents a more regular structure made of elongated parallel axons with a quasi-circular cross section (Støverud et al. 2012). In addition, the blood vessel system runs through the parenchyma to supply oxygen and nutrients. This simplified description of the brain is not meant to be exhaustive but highlights that the brain is a multiphasic material (Ehlers and Wagner 2013). Nevertheless, as pointed out by Tavner et al. (2016), the correct mathematical framework to model the brain parenchyma is still a controversial subject which depends on the specific phenomenon studied.

In this work, since the blood vessels occupy less than 3% of the total volume (Duval et al. 2016), we describe the white matter as a biphasic continuum in which the axons represent the solid phase which is immersed in the ECS which constitutes the fluid phase. Under the hypotheses of incompressible fluid and very low Reynolds number, the convective flux through the axons can be described by means of Darcy’s law, which relates the pressure loss across a porous medium with its average velocity according to the hydraulic permeability (Dullien 2012; Kim et al. 2012; Støverud et al. 2012; Ehlers and Wagner 2013). The latter depends only on the porous media geometry and the fluid properties (Yazdchi et al. 2011), and it can be computed in three different ways:

(i) Experimentally: numerous experimental techniques have been developed and described in the geotechnical literature (Türkkan and Korkmaz 2015), but to the best of our knowledge, only a limited number of studies can be found concerning human tissues (Swabb et al. 1974; Netti et al. 2000; McGuire et al. 2006; Franceschini et al. 2006).
  In particular, Swabb et al. (1974) conducted the first in vitro experimental campaign which aimed to infer the hydraulic permeability of hepatocarcinoma, the most common liver cancer. Netti et al. (2000) performed confined compression test on slices of freshly excised tissue belonging to four tumour lines. Then, they estimated the permeability fitting the experimental data with a poroviscoelastic model. McGuire et al. (2006) followed a similar approach implanting three tumour lines in mice. Then, after the injection of a controlled flow of Evans blue-labelled albumin in the centre of the cancerous tissue, the latter was excised and sliced. Finally, the albumin distribution was fitted by means of Darcy’s law for unidirectional flow in an infinite region around a spherical fluid cavity. Franceschini et al. (2006) conducted an extensive and comprehensive work in which they performed several types of mechanical tests on human brain samples within 12 h of death. Without entering into details, we will just focus on the permeability extraction. They performed an oedometric test on 12 cylindrical specimens harvested in the parietal lobe. The average ratio between initial and final specimen’s shortening under a loading step, namely consolidation ratio, was depicted as a function of time. These data were fitted according to Terzaghi's theory thus allowing to infer the permeability. Despite the works cited above being extremely valuable, they are affected by two important limitations. First, the permeability is not measured directly, but it is inferred from a model which is based on certain assumptions. and second, the hydraulic permeability decreases with time post-mortem and its estimation is therefore affected by the exact time measurements have taken place (Tavner et al. 2016).
(ii) An alternative methodology with respect to the experimental one is using the Kozeny–Carman equation which relates permeability to other geometrical parameters such as porosity and specific surface; for details, the reader can refer to Xu and Yu (2008) and citation therein. However, the major drawback of the analytical approach is that it is only suitable for simple and regular geometries but cannot be applied to complicated structures such as the white matter.
(iii) Finally, in the numerical approach, Navier–Stokes equations are solved to obtain the permeability under some hypotheses. It has been proven to be a powerful tool to analyse random arrangements of fibres as shown in Hitti et al. (2016), Nedanov and Advani (2002) and Takano et al. (2002) or other porous media (Pinela et al. 2005; Kolyukhin and Espedal 2010; Dias et al. 2012; Zeng et al. 2015; Eshghinejadfard et al. 2016). For example, Hitti et al. (2016) computed the permeability of a unidirectional disordered fibres array with constant diameter by first assessing the velocity and the pressure fields of the convective flow through them. Then, by means of an homogenisation method, they obtained the permeability of the whole domain.

In this paper, we develop an approach that for the first time applies numerical techniques to the study of the brain microstructure. The brain geometry and spatial organisation are considered to describe the inter-axons convective flux.

We present an outward packing method to create a bidimensional random geometry based on the ADD provided by Liewald et al. (2014) that ensures a ECS volume fraction and a ECS width in the physiological range (Syková and Nicholson 2008). Moreover, a spatial analysis, by means of Ripley’s k-function (Hansson et al. 2013; Marcon et al. 2013), is conducted to guarantee that the overall geometrical organisation is consistent with the one of the experimental data. Then, a computational fluid dynamics (CFD) model is implemented within the commercial software ANSYS (ANSYS, Lebanon, NH) to compute the white matter hydraulic permeability which will be compared with other data available from the relevant literature.

## Materials and methods

### Dataset

In the study conducted by Liewald et al. (2014), the authors measured the inner diameter of myelinated axons in three anatomical structures namely corpus callosum (CC), superior longitudinal fascicle (SF) and uncinate/inferior occipitofrontal fascicle (IF). Their analysis was performed on three human brains and a monkey brain. Since the first ones underwent a late fixation that could lead to degradation of cellular material and a reduction of hydraulic permeability as pointed out by Tavner et al. (2016), we used the ADD of the monkey which guaranteed an higher fixation quality. Moreover, since we are interested in the external diameter, we added the average myelin sheath width, measured by Liewald et al. (2014), to the ADD.

### Brain-like geometry

The first objective was to design a geometry which could mimic the white matter structure and spatial organisation. Therefore, we created a two-dimensional random disordered fibres packing with a circular cross section which met four important geometrical requirements that drive the convective flux in the extra cellular space: axon diameter distribution, ECS volume ratio, ECS width and spatial organisation.

**Fig. 1:**
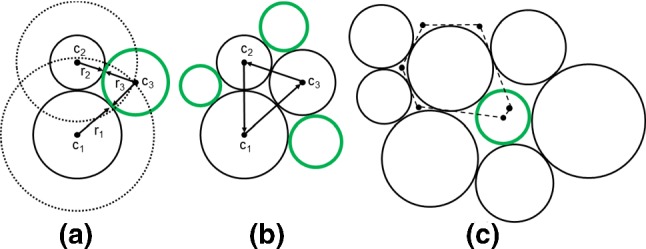
Discs generation algorithm: **a** given two discs with radius $r_1$ and $r_2$ and centred at $c_1$ and $c_2$, respectively, the centre $c_3$ of the new disc (green) with radius $r_3$ is given by one of the two intersections of the dotted discs with radius $r_1+r_3$ and $r_2+r_3$ centred at $c_1$ and $c_2$, respectively; **b** the first three discs form the initial propagation front, a new disc is added on the right side of each arrow; **c** in the second part of the algorithm, new discs are added at the skeleton branch points (black dot) if their diameter is comprised in the ADD

The generation algorithm was based on the closed form advancing front approach presented by Feng et al. (2003), but with a main difference. This work introduces an optimisation phase which pushes the ECS volume fraction at a lower level with respect to the previous method in order to meet the physiological requirements. All the algorithm here presented was developed in the environment provided by MATLAB:

1. The user specifies the total number of fibres, which are represented by discs of varying diameters in our two-dimensional representation, and the desired ADD and ECS volume ratio. Then, he indicates the shape of the domain inside which he wants to insert the discs, e.g. a square or a rectangle, with a certain ratio between adjacent edges. The initial domain area and its boundaries are computed from the sum of each disc area using simple geometrical arguments and calculations. This initial area is not big enough to host all the discs because it does not consider the empty spaces. Therefore, the area increases iteratively until all the discs have found space.
2. The algorithm is based on the following geometrical consideration: given a couple of discs, it is always possible to add a third one which is tangent to both of them if the distance between the first two is less than the diameter of the third; this is schematically depicted in Fig. 1a. Figure 1b shows the polygon formed by the disc centres which constitutes the front along which the generation algorithm propagates. Each new disc is accepted if it is contained inside the domain boundaries and if no overlapping with the other discs occurs.
3. Once all the discs are placed in the domain, the ECS volume ratio is computed as the ratio between the void spaces between the discs and the total area. The outcome of this first part of the algorithm is a highly packed structure with an ECS volume ratio of about 0.22.
4. However, as stated by Syková and Nicholson (2008), the ECS volume ratio can reach a minimum of 0.15 in the brain; for this reason, we implemented an optimisation algorithm which fills the empty spaces in the structure. It could be summarised in four additional steps:
  (i) The original geometry is converted in a black and white image to allow morphological analyses, which are a collection of nonlinear operations related to the shape or morphology of features in an image (Patil and Bhalchandra 2012).
  (ii) The subsequent step is the skeletonisation that, starting from a black and white image, uses the iterative thinning algorithm to reduce all the objects to lines, without changing the essential structure of the image (Haralick and Shapiro 1992). The branch points of the skeleton represent the location where the distance between close discs is maximised. In other words, they are the best locations where it is possible to add new discs as can be appreciated in Fig. 1c.
  (iii) Even in this case the new disc is accepted if its diameter is comprised in the range of the ADD previously defined.
  (iv) The process continues iteratively until reaching the minimum physiological ECS volume ratio.
5. Finally, the desired porosity is achieved by means of a shrinking technique as described in Hitti et al. (2016). It is easy to understand that the discs shrinking affects the desired ADD. However, for the physiological porosity range, which does not exceed 0.3, the shrinking produces a decrease in the axons diameter of only 2.5% which could be considered negligible.

It must be noticed that the second part of the algorithm, where the empty spaces are filled with discs, changes the ADD. Indeed, since the void spaces are small, they are more likely occupied by the discs with a smaller diameter. Nevertheless, this limitation could be considered negligible as discussed in “Appendix 1”.

### Spatial distribution analysis

To compare the permeability evaluated both within the same ADD and between different ADDs as a function of ECS volume ratio, it was necessary to ensure that the spatial organisation of every geometry was consistent. Therefore, the ability of the algorithm described in Sect. 2.2 to create random arrangements of axons was quantified by means of Ripley’s function (Ripley 1976). The axon centres represent a spatial point process, see the contribution by Diggle (2003) for details, and Ripley’s function was used to differentiate between: (1) aggregation, where the points tend to stay close to other points, (2) inhibition where the points form a regular pattern and (3) complete spatial randomness (CSR) where the points do not follow any specific rule (Jafari-Mamaghani 2010; Lang and Marcon 2010; Marcon et al. 2013).

Moreover, we compared the model spatial organisation with the experimental one analysing the transmission electron microscopy (TEM) images provided by Liewald et al. (2014). Therefore, as a preliminary step, we manually segmented the microscopy images and computed the centroids for each anatomical structure (Gopi 2007).

Ripley’s function is defined as:

1 $$\begin{aligned} R(t)=\lambda ^{-1} E \end{aligned}$$

where $\lambda$ is the number of points per unit area, namely the intensity, and *E* is the number of extra points within a distance *t*, which is the distance scale considered, of an arbitrary point (Ripley 1976). For a homogeneous Poisson process that characterises the CSR:

2 $$\begin{aligned} R(t)=\pi t^2 \end{aligned}$$

given the location of all points within a domain, the equation below describes how to compute *R*:

3 $$\begin{aligned} R(t)=\lambda ^{-1} \sum { \sum {w(l_i,l_j)^{-1}}\frac{I(d_{ij}<t)}{N}} \end{aligned}$$

where $d_{ij}$ is the distance between the *i*th and *j*th points, *N* is the total number of points and *I*(*x*) is a function whose value is 1 if the distance between the *i*th and *j*th points is less than *t* and otherwise is zero. Finally, $w(l_i,l_j)$ provides the edge correction to minimise the effects that arise because points outside the boundary are not counted (Dixon 2002). Usually, it is convenient to linearise the R-function as:

4 $$\begin{aligned} L(t)=\root \of {\frac{R(t)}{\pi }} \end{aligned}$$

because the *L*-*function* plot for a CSR distribution is a simple line with an angular coefficient equal to 1 and passing from the origin. On the contrary, for clustering and inhibition the angular coefficient is higher and lower than 1, respectively. Thus, it is easier to show the deviation from CSR and the length scale at which it occurs (Dixon 2002; Hitti et al. 2016; Chen and Sarntinoranont 2007).

### Brain convection model

In the brain, the axons represent the solid phase of the white matter which is immersed in the ECS. As well as the other cells, they could be modelled as a soft tissue but a unique answer on which constitutive model is more appropriate is still missing. For example, for Støverud et al. (2012) the solid phase behaves as an isotropic linear elastic material, whereas Ehlers and Wagner (2013) used a hyperelastic model. On the other hand, other authors stated that if the flow rate is very low, the deformation provoked by the fluid–structure interaction can be considered negligible and therefore, it is possible to safely model the axons as a rigid material (Kim et al. 2010,2012 ; Raghavan and Brady 2011). Since the interest of this study is to infer the permeability in a quasi-static condition (creeping flow), we follow the latter approach and we model the solid phase as a rigid porous media, whose continuity equation is:

5 $$\begin{aligned} {\mathbf {\nabla }} \cdot {\mathbf {v}}=0 \end{aligned}$$

where ${\mathbf {v}}$ is the fluid superficial velocity.

The well-known Darcy’s law is a macroscopic relation between the pressure loss $\nabla p$ and $\tilde{{\mathbf {v}}}$ which is the velocity through the pores averaged on the fluid volume $V_f$ (Eqs. 6 and 7, respectively)

6 $$\begin{aligned} \tilde{{\mathbf {v}}}=\ & {} \frac{{\mathbf {k}}}{\mu } \nabla p \end{aligned}$$

7 $$\begin{aligned} \tilde{{\mathbf {v}}}= & {} \frac{1}{V} \int _{V_f}{ {\mathbf {v}} \hbox {d}V} \end{aligned}$$

where ${\mathbf {k}}$ is the permeability of the porous media, $\mu$ is the viscosity of the fluid ($10^{-3} \, \hbox {Pa}\,\hbox {s}$) (Jin et al. 2016), *V* and $V_{\mathrm{f}}$ are the total and fluid volume, respectively (Yang et al. 2014; Hitti et al. 2016). The superficial velocity though the pores was computed solving the Navier–Stokes equations by means of the finite element method (FEM) software ANSYS (ANSYS, Lebanon, NH) with semi-implicit methods for pressure linked equations (SIMPLE) (ANSYS 2017). A no slip condition was set on each wall and the conduct length was designed to have a fully developed flow before the porous zone. The boundary condition at the inlet (velocity inlet 0.0024 m/s) was chosen to have a very low Reynolds number $Re \approx 10^{-3}$ to respect Darcy’s law hypothesis and to have a velocity close to the one that is usually used in CED intervention (Barua et al. 2013, 2014). A zero pressure was applied at the outlet to reproduce the conventional experimental conditions for measuring hydraulic permeability (Yazdchi et al. 2011; Truscello et al. 2012; Hitti et al. 2016).

### RVE size determination

According to Drugan and Willis (1996) an RVE is: “the smallest material volume element of the composite for which the usual spatially constant (overall modulus) macroscopic constitutive representation is a sufficiently accurate model to represent the mean constitutive response”. However, as stated by Du and Ostoja-Starzewski (2006), a lot of studies are based on the existence of a so-called RVE, but only a few of them have quantitatively determined its size with respect to the microheterogeneity. As previously described in Sect. 2.2, the ECS volume ratio can range between 0.18 and 0.3; however, we decided to limit our study to geometries with the highest value for the following reason. Since the space between each axon is proportional to the ECS volume ratio, choosing a value equal to 0.3 leads to a geometry with a larger ECS width. This characteristic is strongly desirable from a computational point of view; indeed, the smaller the inter-axons space is, the more the meshing process becomes challenging and the simulation dramatically more time-consuming.

In this work, we created 6 (*n*) random structures for each ADD (CC, SF and IF). The mean permeability ${\bar{k}}$ and the standard deviation $\sigma$ were computed for each brain zone as a function of the RVE size.

8 $$\begin{aligned} {\bar{k}} =\ & {} \frac{1}{n} \sum \limits _{i=1}^nk_i \end{aligned}$$

9 $$\begin{aligned} \sigma =\ & {} \root \of {\frac{1}{n-1} \sum \limits _{i=1}^n{(k_i-{\bar{k}})}} \end{aligned}$$

**Fig. 2:**
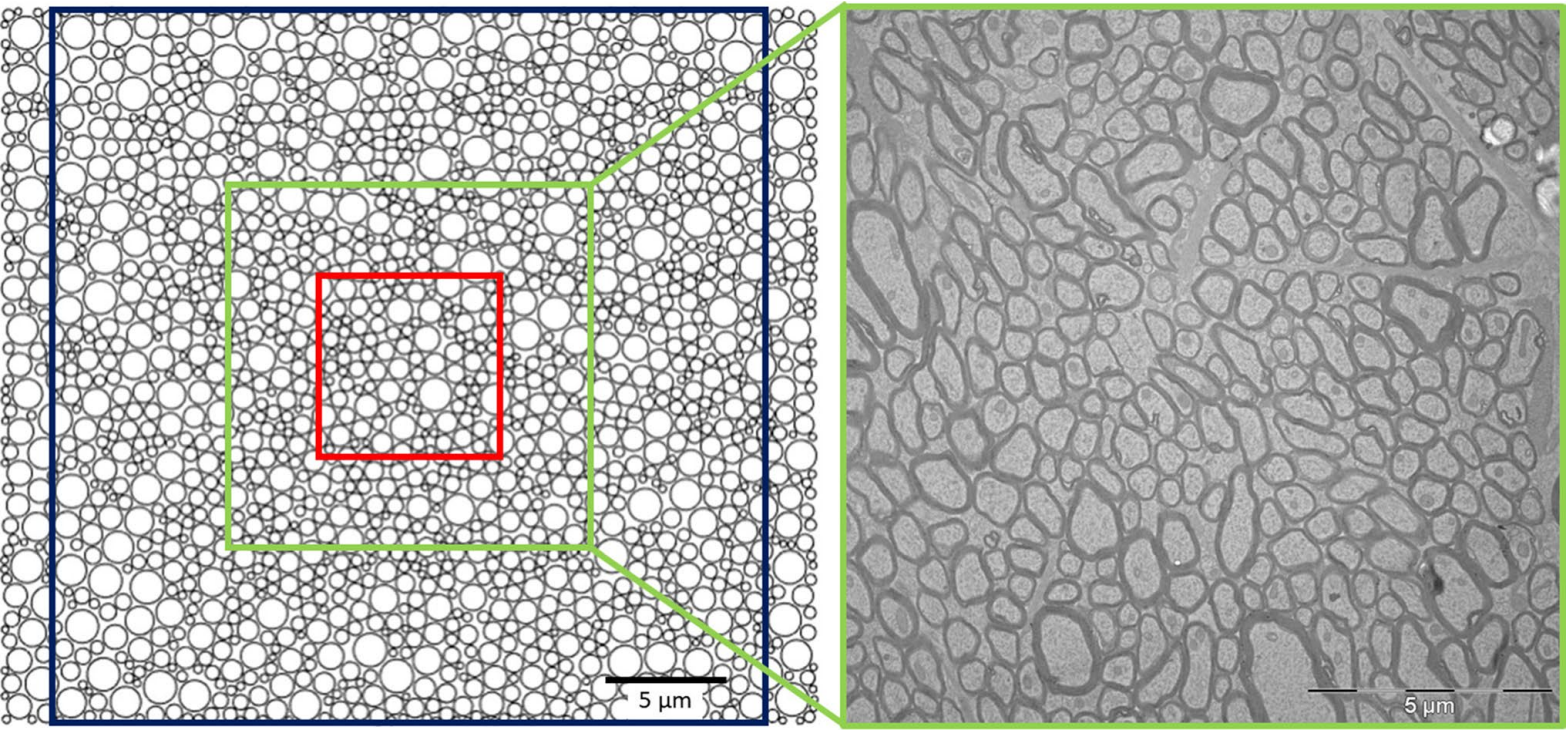
On the left: each model geometry was divided in 20 square RVEs whose edge length is a fraction of the porous media height. The picture shows 5/20 (red), 10/20 (green) and 20/20 (blue); on each RVE the permeability was computed by means of Darcy’s law. On the right: TEM image of the SF, with courtesy of Prof. Dr. Almut Schüz (Liewald et al. 2014)

The RVEs size was determined dividing the height of each model geometry by 20 as shown in Fig. 2 which also depicts a comparison between the model geometry and a TEM image belonging to the SF. However, only the first 16 RVEs were considered for the calculation as a consequence of the *channelling effect* described in Nield and Bejan (2013) which rises at the walls. A detailed explanation can be found in “Appendix 2”.

## Results

### Geometry

**Fig. 3:**
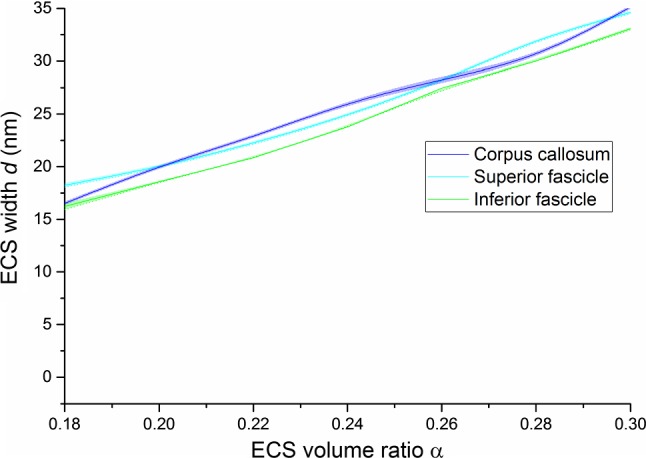
The ECS width is represented as a function of the ECS volume fraction for CC, SF and IF. The ECS width increases in quasi-linear way from a minimum of 16 to a maximum of 35 nm

Figure 3 shows the relationship between two geometrical parameters that are fundamental in determining the fluid dynamics within a porous media, namely, the ECS volume ratio $\alpha$ and the ECS width *d*. The latter has been identified by Syková and Nicholson (2008) as an “atmosphere” surrounding every axon which can be quantified by the following equation:

10 $$\begin{aligned} d =\frac{V_{\mathrm{axon}}}{S_{\mathrm{axon}}}\frac{\alpha }{1-\alpha } \end{aligned}$$

where $V_{\mathrm{axon}}$ and $S_{\mathrm{axon}}$ are the average axon volume and surface area for an ideal thin slab of length equal to 1 $\upmu$m. As depicted in Fig. 3, the ECS width in our model increases in a quasi-linear fashion with the ECS volume ratio from a minimum of 16 nm to a maximum of 35 nm which is comparable with the range identified by Syková and Nicholson (2008). The minimum ECS volume ratio that we were able to reach with our method was equal to 0.18, which is very close to the experimental minimum value of 0.15 (Syková and Nicholson 2008).

**Fig. 4:**
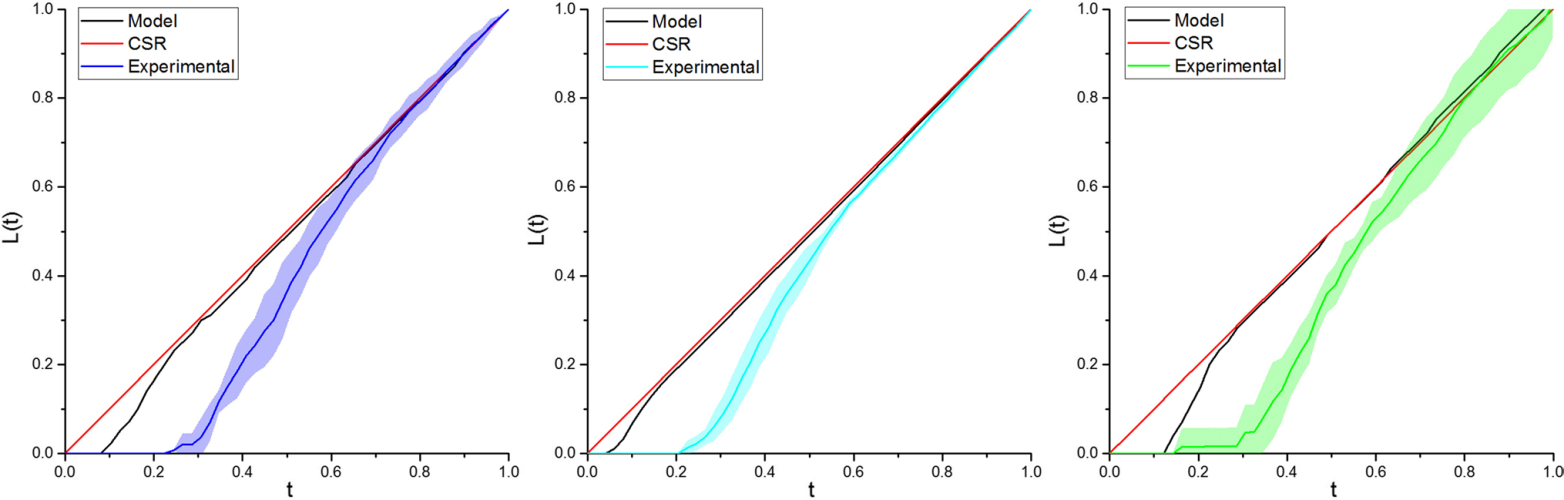
In each graph, it is possible to appreciate the comparison between the $L-function$ under ideal CSR hypothesis (red line), the $L-function$ obtained with model described in Sect. 2.2 and the $L-function$ computed on the TEM images of CC (blue), SF (light blue) and IF (green)

Figure 4 depicts the results of Ripley’s function analysis applied to the TEM images and to the geometry generated through the algorithm described in Sect. 2.2. Moreover, it is possible to compare them with the ideal case of CSR. We can observe that in all the anatomical structures the spatial organisation of both real and model axons is almost coincident to the CSR as we approach the final part of the curve. It should be noted that there is an initial discrepancy between the experimental and the model trend. However, this could be easily explained since the number of axons for each image was significantly lower than the one in the model. Therefore, the presence of big axons in the TEM images strongly affects the analysis, whereas their effect is mitigated in the model geometries. Nonetheless, for *t* equal to 1 which is a normalised value corresponding to the 25% of the image length as suggested in Jafari-Mamaghani (2010), both experimental and model data converge to CSR.

### Grid sensitivity analysis

**Fig. 5:**
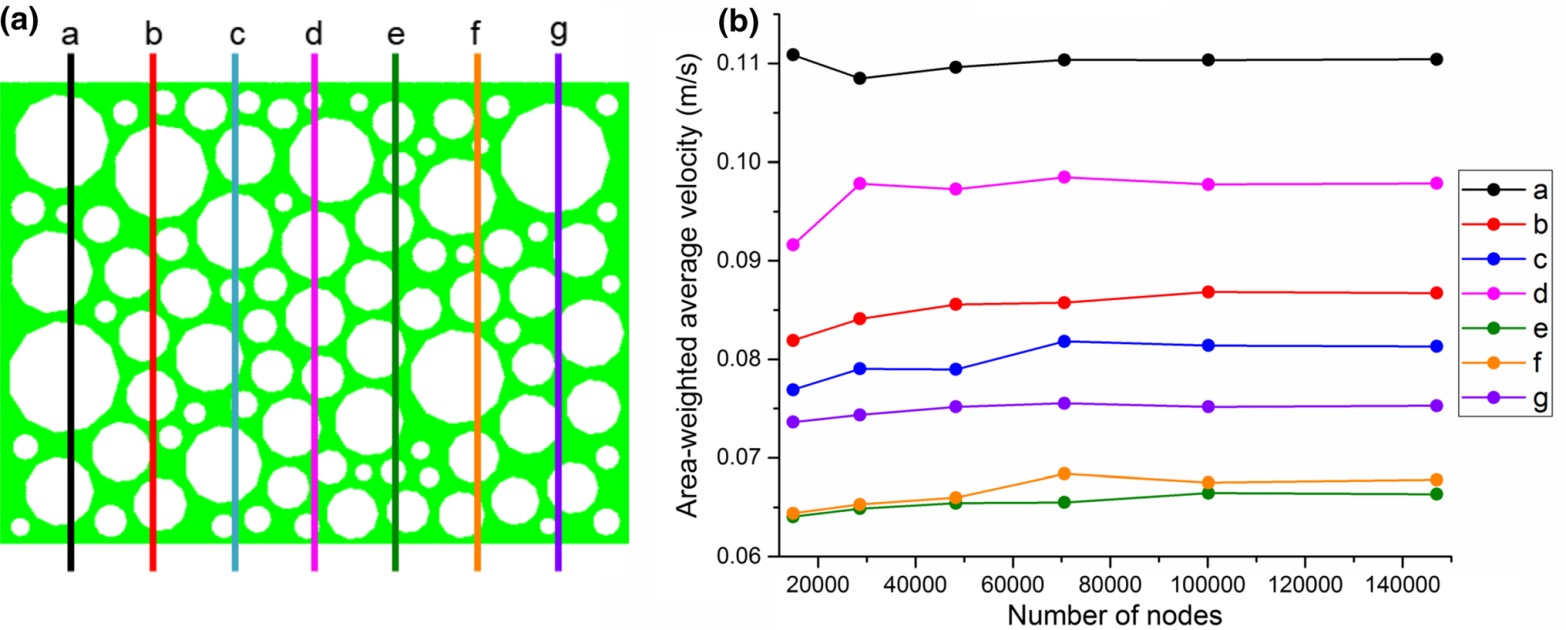
**a** Geometry used to perform the mesh sensitivity analysis, also showing the lines along which the velocity has been averaged. **b** Effect of the grid resolution on the area-weighted average velocity is shown. Note that convergence is reached after about 100,000 nodes

The first important step is to perform a grid sensitivity analysis to find the correct trade-off between the discretisation error reduction and the cost of the simulation in terms of computational time (Montazeri and Blocken 2013). The grid resolution depends on different parameters; we varied separately the maximum face size allowed for each cell and the edges’ discretisation in the porous zone (ANSYS 2017). We compared 6 grids with an increasing number of nodes, from a coarse one, characterised by 14,862 nodes and an average element size of $0.16 \,\times \,10^{-2} \, \upmu \hbox {m}^2$, to a finer one corresponding to 153,496 nodes and $0.015 \,\times \, 10^{-2} \, \upmu \hbox {m}^2$ average element size. In Fig. 5, it is possible to appreciate the geometry used for the grid sensitivity analysis and the lines along which the velocity has been computed, and the results of the analysis are shown on the right. The independence of the average velocity from the grid resolution is achieved for a number of nodes close to $10^5$. Indeed, the percentage error between the grids with 100,155 and 147,016 nodes ranges between 0.08 and 0.4%, which can be considered negligible (Montazeri and Blocken 2013). Therefore, further analysis was performed following the discretisation features of the 100,155 nodes grid which has been proven to assure high accuracy and adequate computational cost. The simulations took 3 h on a workstation with a i7-6800K 6 cores 3.60 GHz CPU and 16 GB of memory.

### RVE size

**Fig. 6:**
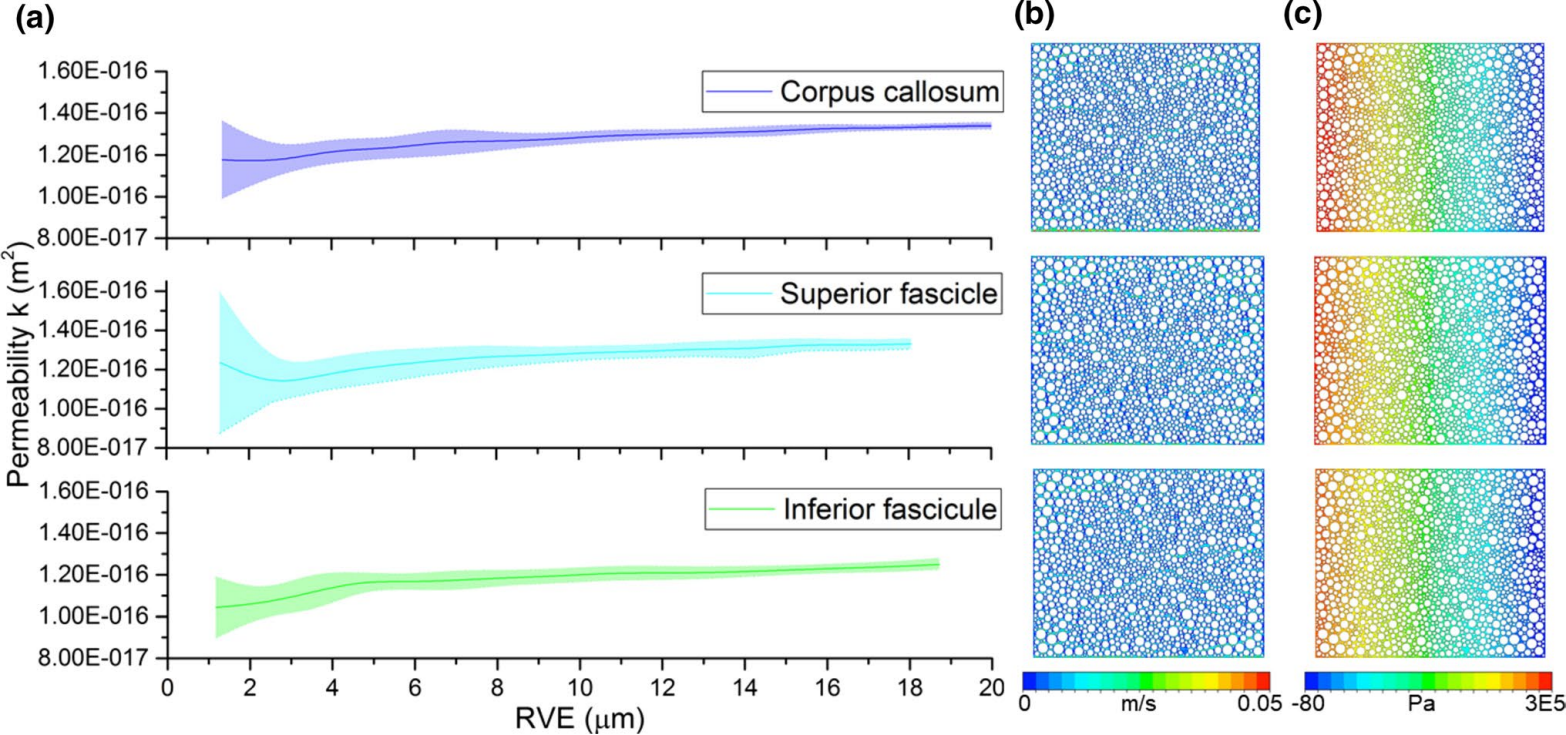
The hydraulic permeability (**a**) in the CC, SF and IF is represented as a function of the RVE size along with the respective velocity (**b**) and pressure contours (**c**)

Figure 6 represents ${\bar{k}}$ as a function of the RVE size for CC, SF and IF. The standard deviation is very high at the beginning when the RVE size is less than 8 $\upmu \hbox {m}$; then, as the RVE size increases, the standard deviation decreases progressively until it becomes two orders of magnitude less than the mean permeability. This is due to the fact that the bigger the area considered for the homogenisation is and the more it is representative of the porous media behaviour. On the other hand, a large area can increase dramatically the computational cost of the simulations. The best trade-off between accuracy and simulation time is identified by the optimal RVE size. In each anatomical area, we found the RVE critical value as the point that satisfies two requirements: the average permeability is constant and the standard deviation becomes a small fraction of the average value. It is worth noticing that the minimum standard deviation is about 2% of the permeability, thus confirming that 6 geometries for each ADD provide a sufficient level of accuracy. The results are summarised in Table 1.

**Table 1:** RVE size and average hydraulic permeability in CC, SF and IF

	CC	SF	IF
RVE $(\upmu \hbox {m})$	17.5	16.8	15.2
${\bar{k}} \, (\hbox {m}^2)$	$1.33 \, \times \,10^{-16}$	$1.32 \, \times \,10^{-16}$	$1.22\, \times \,10^{-16}$

Furthermore, Fig. 6 shows examples of velocity and pressure contours for each ADD. In each geometry, the flow paths as well as the maximum velocity is very similar since the average ECS width, which drives the convective flux in CC, SF and IF, is comparable. Moreover, the pressure field decreases linearly along the porous media with an overall pressure drop of about 30,000 Pa.

### Comparison with previous studies

In the literature, there exist a few studies concerning hydraulic permeability in human tissues, which report a wide range of values. Table 2 lists three of the major experimental papers where the authors used different types of tissue (Netti et al. 2000; Swabb et al. 1974; Franceschini et al. 2006). The obtained results vary significantly and cover a range of three orders of magnitude. This suggests a strong correlation between permeability and histological features. Our results are well within the experimental range.

**Table 2:** Experimental studies on hydraulic permeability with several types of tissues

Tissue type	Permeability $(\hbox {m}^2)$	Researchers
Hepatic neoplastic tissue in vitro	$3.1\, \times \,10^{-17}$	Swabb et al. (1974)
Hepatic neoplastic tissue in vivo	(2.9–8.4)$\, \times \,10^{-18}$	Swabb et al. (1974)
MCaIV murine mammary carcinoma	$1.86\, \times \,10^{-15}$	Netti et al. (2000)
LS174T human colon adenocarcinoma	$3.37\, \times \,10^{-16}$	Netti et al. (2000)
U87 human glioblastoma	$4.87\, \times \,10^{-16}$	Netti et al. (2000)
HSTS 26T human soft tissue sarcoma	$6.9\, \times \,10^{-17}$	Netti et al. (2000)
Human brain tissue	$2.47\, \times \,10^{-17}$	Franceschini et al. (2006)

## Discussion

The relevant literature concerning fibrous porous media has seen many attempts to describe the hydraulic permeability of unidirectional fibres; the models can be roughly divided in ordered and disordered where the analytical or numerical approach has been followed, respectively. In the former category, an analytical relationship between hydraulic permeability and porosity can be established according to the fibres packing (triangular, square, hexagonal) as described by Gebart (1992) and Tamayol and Bahrami (2009). On the contrary, in the second category, computational methods have been used to understand how permeability is influenced by other geometrical factors such as the mean nearest inter-fibres distance and the degree of disorder (Chen and Papathanasiou 2007, 2008; Hitti et al. 2016). Although the contributions of the researches cited above are valuable and underline the importance of the geometry on the overall behaviour of the porous media, they use a population of fibres with the same diameter which is not the case of the white matter as explained in Sect. 2.1. Therefore, the presence of a geometry which is able to mimic the main geometrical characteristics of the white matter is fundamental to model effectively the flow through the axons. In Sect. 3.1, we demonstrated how we achieved this task implementing a model geometry in which the main histological features of the white matter are considered. Indeed, the ECS volume fraction covers 87% of the physiological range. Moreover, the ECS width is in very good agreement with the experimental data presented in the literature, also considering the inter-species variability, since they analysed murine brain, and the differences between grey and white matter (Nicholson et al. 2011; Ohno et al. 2007; Nicholson and Hrabětová 2017; Syková and Nicholson 2008).

Furthermore, we exploited Ripley’s function to inquire the spatial organisation as depicted in Fig. 4. Although a comprehensive analysis that covers the entire parameter space is out of the scope of this work, the randomness analysis performed on either the experimental images and our model shows a behaviour which is ascribable to CSR. Moreover, assessing the spatial organisation of a porous media and ensuring that it is homogeneous along all the length scale considered is fundamental in all the studies that aim to estimate the correct size of an RVE (Hitti et al. 2016).

The sensitivity analysis conducted on the grid resolution allowed us to obtain accurate results as well as a feasible computational times for a challenging geometry.

The permeability of each ADD was computed on RVEs of increasing size. The results illustrated in Fig. 6 and Table 1 show outcomes concerning both the RVE critical size and the permeability values which were similar in the cases examined. This is probably due to the fact that even if we are considering three different anatomical structures, their ADD as well as the ECS width are very similar, thus producing a comparable effect on the fluid flow as suggested also by Chen and Papathanasiou (2008) in their discussion on the mean nearest inter-fibres distance. On the other hand, comparing our results with data presented in the literature has proven to be a more difficult task since a very small amount of experiments have been conducted. The work which is closest to our study is that performed by Franceschini et al. (2006), who computed a permeability value which is slightly lower than ours. However, it must be noticed that there are four important differences to take into account. Firstly, there is an inter-species variability, as suggested by Abbott (2004), since we are analysing a monkey brain instead of a human one. A second factor to consider is that the permeability is not a direct measure but it is inferred from a model which is based on simplifying hypotheses and, for example, does not consider non-circular axons and deviation from collinear bundles, which would both contribute to lower the permeability of the tissue. Third, the results obtained by Franceschini et al. (2006) are an average between brain samples excised in both grey and white matter, whereas we limit our study to white matter. Finally, the average ECS volume ratio in the brain is about 0.2 (Syková and Nicholson 2008), whereas we used the maximum value of 0.3 for the reasons explained in Sect. 2.5. Since the ECS volume fraction is directly related to permeability, this contributes to the lower value obtained by Franceschini et al. (2006).

Nevertheless, our results are in good agreement with the experimental data if compared to the range of values presented in the literature and represent the first attempt to estimate the permeability with a numerical approach which starts from the white matter microstructure. The method presented in the present contribution opens the possibility to further extend the study incorporating more images belonging to normal or pathological subjects, thus allowing to create a specific database for the permeability of brain tissue matter.

**Fig. 7:**
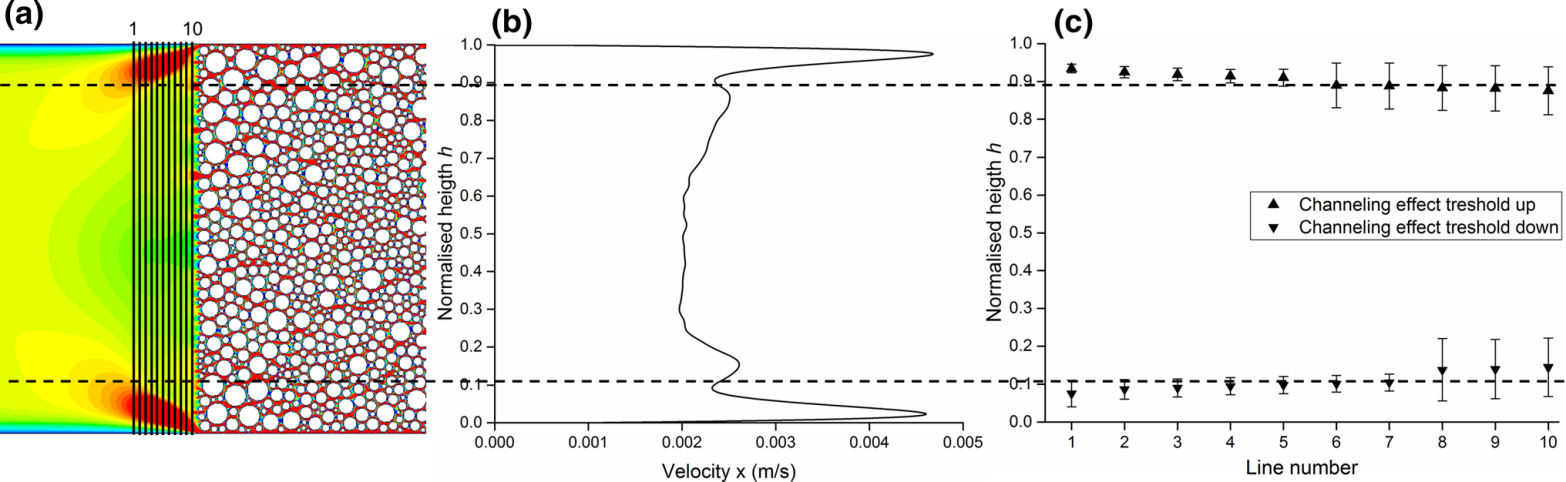
**a** Velocity contour before the porous media, the *channelling effect* is clearly visible near the walls. The black lines indicate the direction along which the velocity profiles have been extracted; **b** average velocity profile for the CC, even in this case the sudden increase in the velocity profile points out the beginning of the *channelling effect* zone; **c** its exact starting points have been determined averaging the position of the first and last local minima between the 6 random geometries of the CC

## Concluding remarks

We presented a novel method to assess hydraulic permeability, starting from the ADD of three white matter anatomical structures. Moreover, we paid particular attention to estimate the RVE size to ensure the reliability of the results obtained. The approach consisted of the following three steps: (1) generation of a random geometry in which the cross-sectional area of the neurons is considered circular. The algorithm created a fibres assembly according to the experimental ADD of CC, SF and IF, offering also the possibility to vary the ECS volume fraction covering almost all the physiological range. (2) Implementation of a CFD model by means of the finite element solver ANSYS to compute the velocity and pressure fields experienced by our model white matter. Furthermore, we conducted a grid sensitivity analysis to ensure high accuracy. (3) Finally, we used these data to compute the hydraulic permeability on different RVEs in order to determine its size.

We found that the RVE size and the hydraulic permeability are slightly different for each anatomical structure suggesting that an RVE characterised by a length scale of about $17 \upmu \hbox {m}$ can be representative of the overall behaviour. Moreover, the permeability values that we found are consistent with the results provided by experimental data available in the literature. Albeit based on simplifying assumptions, we believe that this work is the first important step towards a combined experimental and computational approach which aims to shed light on fundamental constitutive parameters to model brain matter. Extensions to three-dimensional domains, consideration of irregular axonal geometries and osmotic pressure, contribution of glial cells and a parametric study on the effect of the ECS volume ratio will constitute the subject of further studies.

## Appendix 1

In Sect. 2.2, we explained that the algorithm to create a brain-like geometry is mainly comprised of two phases. In the first phase, the fibres are randomly arranged respecting a prescribed ADD, and the minimum ECS volume ratio reachable in this phase is about 0.22. In the second phase, whose objective is to minimise the ECS volume ratio, the empty spaces are filled with other fibres whose diameter is comprised in the range of the ADD. Since the axons with a small diameter are more likely to find room between the others, the ADD is more skewed towards them with respect to the original one. That results in a median diameter which goes from the 0.34 $\upmu \hbox {m}$ of the original ADD to the 0.3 $\upmu \hbox {m}$ of the skewed ADD. To quantify the effect of this limitation on the permeability calculation, we created a geometry respecting the ADD of the CC. Then, applying the shrinking method described in Sect. 2.2, we reached the desired ECS volume ratio equal to 0.3.

We computed the permeability on an RVE of 17.5 $\upmu \hbox {m}$ as suggested by the results reported in Sect. 3.3 obtaining a final value equal to $1.4 \, \times \,10^{-16} \, \hbox {m}^2$ which is 5% higher than the one presented in Table 1. In conclusion, our generation algorithm, on the one hand, introduces a very small error, and, on the other hand, allows analysing almost all the physiological range of ECS volume fraction. We believe that the increased flexibility obtained by the proposed algorithm and its fidelity in reproducing realistic ECS volume fractions greatly overcome the potential error introduced in the computation of permeability, and therefore, we considered this limitation acceptable.

## Appendix 2

In the attempt of filling a volume or an area with solid particles, a common issue usually rises in the proximity of the walls. Indeed, here, the particles find it harder to pack together, with respect to the inner zones of the porous media, because of the presence of the walls. Therefore, the free space volume fraction increases; for an analytical description of this phenomenon, the reader can refer to the work by Nield and Bejan (2013).

As it is easy to imagine, the volume fraction increase brings, as a consequence, the augmentation of the volume of fluid flowing near the walls as well as the average velocity, and this is evident in Fig. 7a. Since this phenomenon, which is known as *channelling effect* (Nield and Bejan 2013), affects the permeability computation, we designed a method to infer and exclude the areas involved.

In each geometry, we extracted the velocity profile along 10 lines in the proximity of the porous zone as indicated in Fig. 7a. The threshold of the *channelling effect* zone can be identified by the anomalous and sudden increase in the velocity profile highlighted in Fig. 7b. Mathematically, this operation means finding the position of the first and last local minima along the normalised height of the channel *h*. Finally, Fig. 7c depicts the position of the upper and lower threshold averaged between the 6 geometries created for the CC. Equivalent results (not shown in this paper) emerged for the other anatomical structures.

Accordingly, the porous media areas corresponding to 10% of the channel height at both ends (top and bottom in Fig. 7a of the computational domain) were excluded from the hydraulic permeability computation.
