# Correction to: A computational fluid dynamics approach to determine white matter permeability

**DOI:** 10.1007/s10237-019-01206-5

**Published:** 2019-08-09

**Authors:** Marco Vidotto, Daniela Botnariuc, Elena De Momi, Daniele Dini

**Affiliations:** 1 Department of Electronics, Information and Bioengineering, Politecnico di Milano, 20133 Milan, Italy; 2 Department of Mechanical Engineering, Imperial College London, London, SW7 2AZ UK; 3 Faculty of Science, University of Lisbon, Campo Grande, 1149-016 Lisbon, Portugal

## Correction to: Biomechanics and Modeling in Mechanobiology (2019) 18:1111–1122 10.1007/s10237-019-01131-7

The article “A computational fluid dynamics approach to determine white matter permeability” written by Marco Vidotto, Daniela Botnariuc, Elena De Momi and Daniele Dini was originally published electronically on the publisher’s Internet portal (currently SpringerLink) on 20 February 2019 without open access.

With the author(s)’ decision to opt for Open Choice, the copyright of the article changed on 8 August 2019 to © The Author(s) 2019 and the article is forthwith distributed under the terms of the Creative Commons Attribution 4.0 International License (http://creativecommons.org/licenses/by/4.0/), which permits use, duplication, adaptation, distribution and reproduction in any medium or format, as long as you give appropriate credit to the original author(s) and the source, provide a link to the Creative Commons license and indicate if changes were made.

The original article has been corrected.

